# (μ_2_-Adipato-κ^4^ *O*,*O*′:*O*′′,*O*′′′)bis­[aqua­(benzene-1,2-di­amine-κ^2^ *N*,*N*′)­chlorido­cadmium]: crystal structure and Hirshfeld surface analysis

**DOI:** 10.1107/S2056989017011677

**Published:** 2017-08-21

**Authors:** Wannur Sofiasalamah Khairiah A. Rahman, J. Ahmad, Siti Nadiah Abdul Halim, Mukesh M. Jotani, Edward R. T. Tiekink

**Affiliations:** a Department of Chemistry, University of Malaya, 50603 Kuala Lumpur, Malaysia; b Department of Chemistry, Kulliyyah of Science, International Islamic University Malaysia, 25200 Kuantan, Pahang, Malaysia; c Department of Physics, Bhavan’s Sheth R. A. College of Science, Ahmedabad, Gujarat 380 001, India; d Research Centre for Crystalline Materials, School of Science and Technology, Sunway University, 47500 Bandar Sunway, Selangor Darul Ehsan, Malaysia

**Keywords:** crystal structure, cadmium, adipic acid, benzene-1,2-di­amine, hydrogen bonding

## Abstract

The full mol­ecule of the binuclear title compound, [Cd_2_Cl_2_(C_6_H_8_O_4_)(C_6_H_8_N_2_)_2_(H_2_O)_2_], is generated by the application of a centre of inversion located at the middle of the central CH_2_—CH_2_ bond of the adipate dianion; the latter chelates a Cd^II^ atom at each end. Along with two carboxyl­ate-O atoms, the Cd^II^ ion is coordinated by the two N atoms of the chelating benzene-1,2-di­amine ligand, a Cl^−^ anion and an aqua ligand to define a distorted octa­hedral CdClN_2_O_3_ coordination geometry with the monodentate ligands being mutually *cis*. The disparity in the Cd—N bond lengths is related to the relative *trans* effect exerted by the Cd—O bonds formed by the carboxyl­ate-O and aqua-O atoms. The packing features water-O—H⋯O(carboxyl­ate) and benzene-1,2-di­amine-N—H⋯Cl hydrogen bonds, leading to layers that stack along the *a*-axis direction. The lack of directional inter­actions between the layers is confirmed by a Hirshfeld surface analysis.

## Abstract 2

In the centrosymmetric binuclear title compound, the Cd^II^ atoms are linked by a μ_2_-adipate dianion; the distorted octa­hedral geometry of the metal ion is defined by a ClN_2_O_3_ donor set.

## Chemical context   

In the +II oxidation state, the 4*d* ^10^ cadmium(II) cation is a favourite of researchers studying coordination polymers/metal–organic frameworks. With the ability to readily coordinate a variety of different donor atoms, *i.e*. both hard and soft donors, and to adopt a range of coordination geometries, a diverse array of structures can be generated. The motivation for studying cadmium(II) compounds in this context, over and above intellectual curiosity, rests primarily with evaluating their photoluminescence properties (Lestari *et al.*, 2014 ▸; Xue *et al.*, 2015 ▸; Seco *et al.*, 2017 ▸).
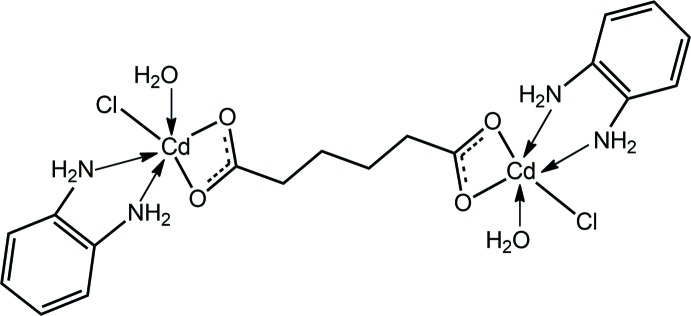


Our inter­est in cadmium(II) structural chemistry is in the controlled formation (dimensionality and topology) of coordination polymers of di­thio­phosphates (^−^S_2_P(O*R*)_2_; Lai & Tiekink, 2004 ▸, 2006 ▸), xanthates (^−^S_2_CO*R*; Tan, Azizuddin *et al.*, 2016 ▸) and di­thio­carbamates (^−^S_2_CN*R*_2_; Chai *et al.*, 2003 ▸), in particular those substituted with hy­droxy­ethyl groups, capable of forming hydrogen-bonding inter­actions (Tan *et al.*, 2013 ▸; Tan, Halim & Tiekink, 2016 ▸). In this connection, we now describe the crystal structure determination and Hirshfeld surface analysis of a cadmium(II) species, (I), with a potentially bridging adipato dianion and an ancillary ligand, benzene-1,2-di­amine, capable of forming hydrogen-bonding inter­actions.

## Structural commentary   

**Figure 1:**
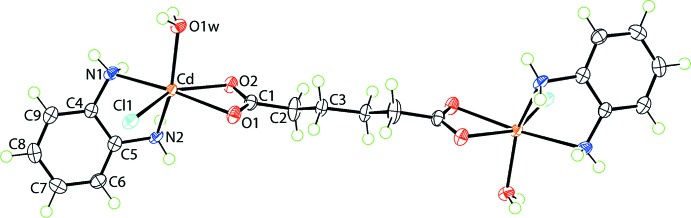
The mol­ecular structure of (I), showing the atom-labelling scheme and displacement ellipsoids at the 70% probability level. The mol­ecule is disposed about a centre of inversion and unlabelled atoms are related by the symmetry operation (−*x*, 2 − *y*, − *z*).

The asymmetric unit of (I) comprises half a mol­ecule of (I), Fig. 1 ▸, with the full mol­ecule generated about a centre of inversion. The key feature of the structure is the tetra-coordinate mode of coordination of the adipato dianion, linking the two Cd^II^ cations. Each carboxyl­ate group forms equivalent Cd—O bonds, the difference in the two bonds being only 0.01 Å, Table 1 ▸. More asymmetry is found in the coord­ination of the benzene-1,2-di­amine ligand with the Cd—N1 bond length being 0.05 Å longer than Cd—N2. This may be traced to the different *trans* effects exerted by the oxygen atoms in that the N1 atom is *trans* to the carboxyl­ate-O1 atom [N1—Cd—O1 = 166.89 (6)°] whereas N2 is opposite to the coordinating water mol­ecule [N2—Cd—O1*W* = 149.12 (7)°]. The coordination geometry is completed by the chloride anion which, owing to the presence of two chelating ligands, occupies a position *cis* to the aqua group. The donor set is ClN_2_O_3_ and defines a distorted octa­hedral geometry.

As might be expected, the four-membered chelate ring formed by the carboxyl­ate group is strictly planar (r.m.s. deviation = 0.0009 Å). There is a twist in the chain of the di­carboxyl­ate ligand with the bond linking the quaternary atom to the aliphatic group being *+ anti-clinal*, *i.e*. the O2—C1—C2—C3 torsion angle is 145.7 (3)° but, *- anti-periplanar* about the central bond, *i.e*. C1—C2—C3—C3^i^ is −177.6 (3)°; symmetry code: (i) −*x*, 2 − *y*, −*z*. There is a distinct kink in the five-membered ring formed by the benzene-1,2-di­amine ligand. This is readily seen in the dihedral angle of 58.57 (7)° formed between the plane through the CdN_2_ atoms and the benzene ring.

## Supra­molecular features   

**Figure 2:**
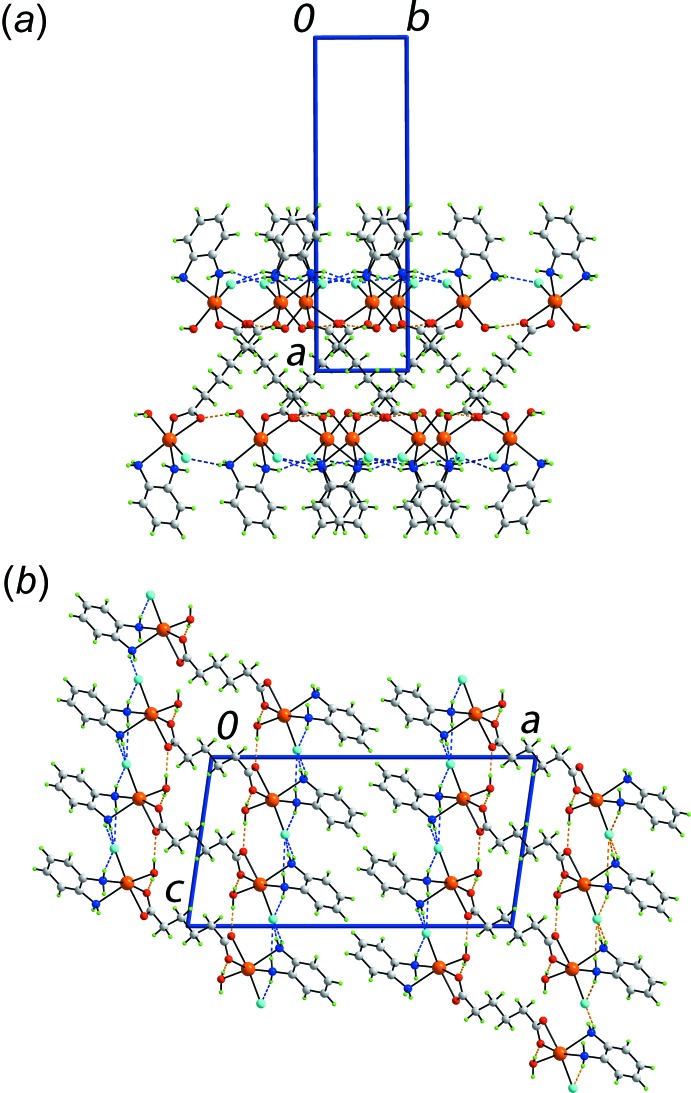
Mol­ecular packing in (I): (*a*) a view of the supra­molecular layer parallel to (100) sustained by water-O—H⋯O(carboxyl­ate) and benzene-1,2-di­amine-N—H⋯Cl hydrogen bonds and (*b*) a view of the unit-cell contents in projection down the *b* axis. The O—H⋯O and N—H⋯Cl hydrogen bonds are shown as orange and blue dashed lines, respectively.

As summarized in Table 2 ▸, all acidic hydrogen atoms in the mol­ecule of (I) are involved in conventional hydrogen-bonding inter­actions. The water-H atoms each form an hydrogen bond with a carboxyl­ate-O atom to form strands propagating along the *b-*axis direction, involving the carboxyl­ate-O1 atoms, and along the *c*-axis direction, involving the carboxyl­ate-O2 atoms. Thereby, a supra­molecular layer is formed parallel to (100), Fig. 2 ▸ *a*. Within this framework are benzene-1,2-di­amine-N—H⋯Cl hydrogen bonds involving all the amine-H atoms. This has the result that each chloride anion accepts four N—H⋯Cl hydrogen bonds and, to a first approximation exists in a flat, bowl-shaped environment defined by a CdH_4_ ‘donor set’. Layers stack along the *a* axis with no directional inter­actions between them, Fig. 2 ▸ *b*. Given this observation, it was thought worthwhile to perform a Hirshfeld surface analysis to probe the mol­ecular packing in more detail. The results of this analysis are discussed in the next section.

## Hirshfeld surface analysis   

**Figure 3:**
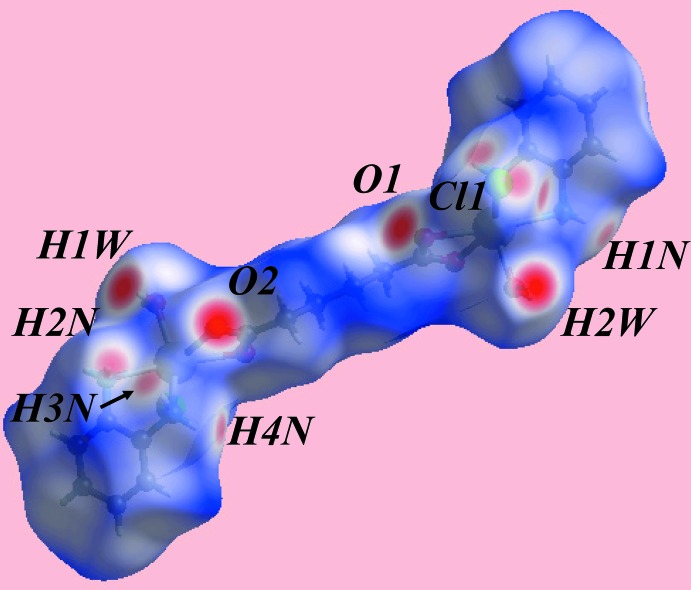
A view of the Hirshfeld surface for (I) mapped over *d*_norm_ in the range −0.597 to +1. 425 au.

**Figure 4:**
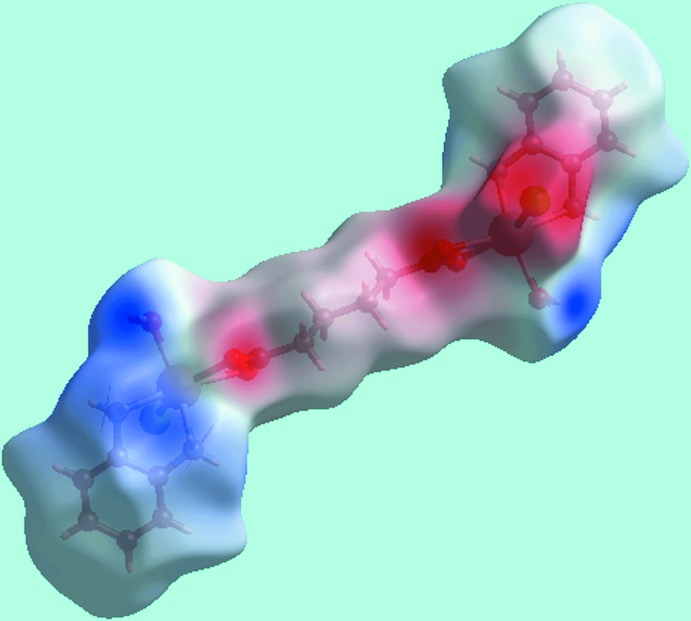
A view of the Hirshfeld surface for (I) mapped over the electrostatic potential in the range −0.164 to +0.204 a.u. The red and blue regions represent negative and positive electrostatic potentials, respectively.

**Figure 5:**
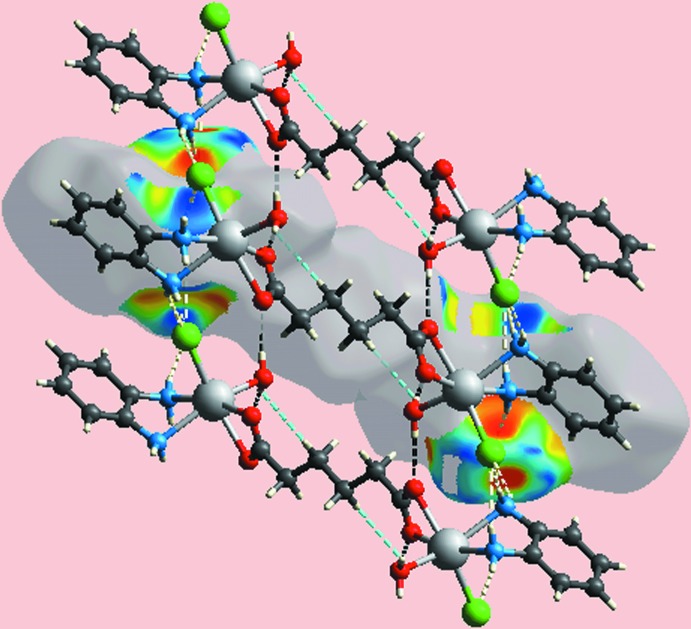
A view of the Hirshfeld surface for (I) mapped with the shape-index property about a reference mol­ecule showing inter­molecular O—H⋯O and N—H⋯Cl contacts as well as short inter­atomic H⋯H contacts as black, white and sky-blue dashed lines, respectively.

The Hirshfeld surfaces calculated for (I) provide further insight into the supra­molecular associations in the crystal; the calculations were performed according to a recent publication (Jotani *et al.*, 2017 ▸). The presence of bright-red spots appearing near water-H atoms, H1*W* and H2*W*, and carboxyl­ate oxygen atoms, O1 and O2, on the Hirshfeld surface mapped over *d*_norm_ in Fig. 3 ▸, result from the O—H⋯O hydrogen bonds between these atoms, Table 2 ▸. The faint-red spots appearing near each of di­amine-hydrogen atoms, H1*N*–H4*N*, and those near the Cl1 atom represent the formation of the four comparatively weak N—H⋯Cl inter­actions. The donors and acceptors of above inter­molecular inter­actions can also be viewed as blue and red regions around the respective atoms on the Hirshfeld surface mapped over the calculated electrostatic potential in Fig. 4 ▸. The immediate environment about a reference mol­ecule within the shape-index mapped Hirshfeld surface highlighting inter­molecular O—H⋯O, N—H⋯Cl inter­actions and short inter­atomic H⋯H contacts is illustrated in Fig. 5 ▸.

**Figure 6:**
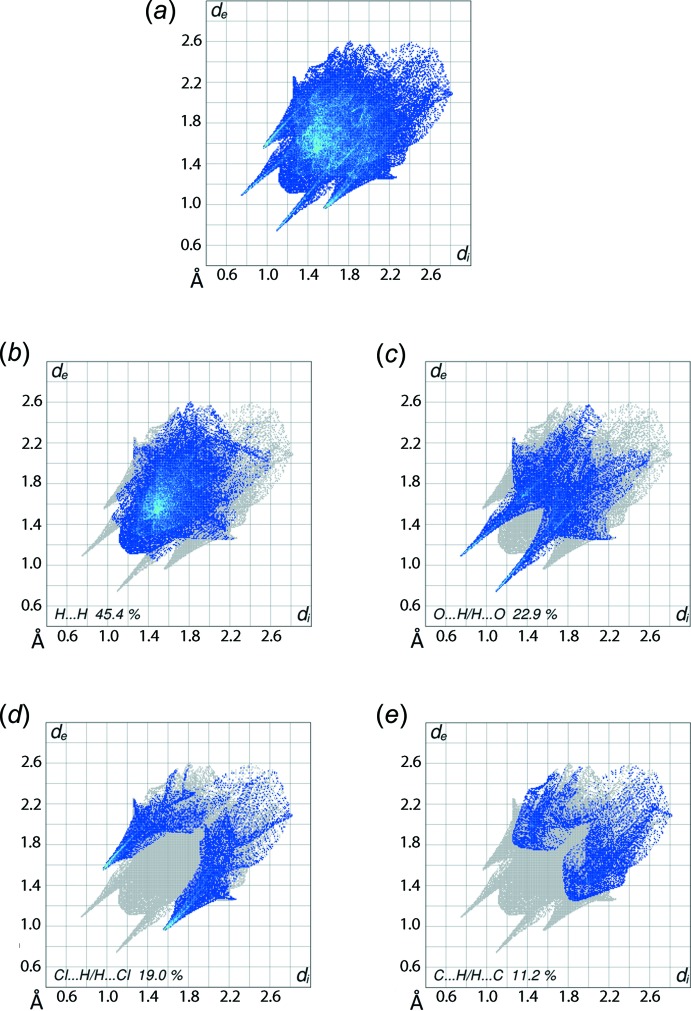
(*a*) The full two-dimensional fingerprint plot for (I) and fingerprint plots delineated into (*b*) H⋯H, (*c*) O⋯H/H⋯O, (*d*) Cl⋯H/H⋯Cl and (*e*) C⋯H/H⋯C contacts.

The overall two-dimensional fingerprint plot, Fig. 6 ▸ *a*, and those delineated into H⋯H, O⋯H/H⋯O,Cl⋯H/H⋯Cl and C⋯H/H⋯C contacts (McKinnon *et al.*, 2007 ▸) are illustrated in Fig. 6 ▸ *b–e*, respectively. The significant contributions from inter­atomic O⋯H/H⋯O and Cl⋯H/H⋯Cl contacts to the Hirshfeld surfaces, see data in Table 3 ▸, result from the involvement of water, di­amine, chloride and carboxyl­ate residues in the inter­molecular inter­actions. The relatively high contribution from these atoms decreases the relative importance of inter­atomic H⋯H contacts, *i.e*. to 45.4%, to the Hirshfeld surface. The presence of a short inter­atomic H⋯H contact between water-H1*W* and methyl-H3*A*, Table 4 ▸, also has an influence upon the mol­ecular packing as shown in Fig. 5 ▸. In the fingerprint plot delineated into H⋯H contacts, Fig. 6 ▸ *b*, this is viewed as the distribution of points at *d*_e_ + *d*_i_ < sum of their van der Waals radii, *i.e*. 2.40 Å. Another short inter-atomic H⋯H contact listed in Table 4 ▸, involving benzene-H8 atoms lying at the surfaces of the layers stacked along the *a* axis appear to have little impact upon the packing. The inter­molecular O—H⋯O and N—H⋯Cl hydrogen bonding are recognized as the pair of spikes at *d*_e_ + *d*_i_ ∼ 1.8 and 2.5 Å, respectively, together with green points within the distributions in Fig. 6 ▸ *c* and *d*, respectively. The points related to short inter-atomic O⋯H contact between water-O1*W* and methyl-H3*A* mentioned above are merged in the plot, Fig. 6 ▸ *c*. It can be seen from the fingerprint plot delineated into C⋯H/H⋯C contacts, Fig. 6 ▸ *e*, that although these contacts make a significant contribution of 11.2% to the dumbbell-shaped Hirshfeld surface due to the presence of benzene-C atoms, the mol­ecular packing results in inter-atomic C⋯H/H⋯C separations longer than van der Waals contact distances, hence they exert a negligible effect in the crystal. The low contribution from other contacts listed in Table 3 ▸ have little effect in the structure due to their large inter-atomic separations.

## Database survey   

A search of the crystallographic literature (Groom *et al.*, 2016 ▸) was undertaken in order to find closely related structures to (I). Reflecting the inter­est in these structures, there were nearly 50 examples with the adipato dianion. In each case, the dianion bridged two Cd^II^ cations *via* chelating inter­actions in all but one example. Often, the di­carboxyl­ate ligand also bridged other Cd^II^ cations, *i.e*. was found to be coordinating in μ_3_- and μ_4_-modes. The most closely related structure in the literature is illustrated in Scheme 2, *i.e*. (II) (Che *et al.*, 2013 ▸).
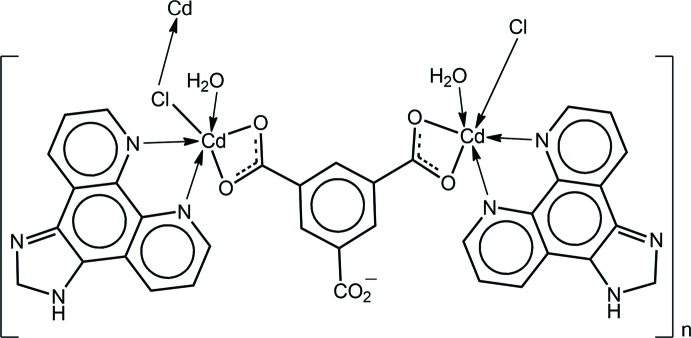


The coordination geometry for one of the independent Cd^II^ atoms in (II), being defined by two carboxyl­ate-O atoms, derived from a tri-anionic μ_2_-benzene-1,3,5-tri­carboxyl­ato ligand, two nitro­gen atoms from a chelating imidazo[4,5-*f*][1,10]phenanthroline ligand, chlorido and water-O atoms resembles that found in (I); this is illustrated on the left-hand side of Scheme 2. The difference between (I) and (II) is that in (II), the chlorido ligand is bridging, leading to a one-dimensional coordination polymer.

## Synthesis and crystallization   

Benzene-1,2-di­amine (0.4324 g, 4 mmol) was slowly added to an aqueous solution (15 ml) of CdCl_2_·2H_2_O (0.4026 g, 2 mmol) resulting in a yellow solution. The mixture was stirred for about 1 h when adipic acid (0.2923 g, 2 mmol) in MeOH (10 ml) was added. The mixture then was stirred for a further 3 h. The resultant solution was reduced and left for crystallization. Brown crystals of (I) were obtained after a few weeks and analysed directly.

## Refinement details   

Crystal data, data collection and structure refinement details are summarized in Table 5 ▸. The carbon-bound H-atoms were placed in calculated positions (C—H = 0.95–0.99 Å) and were included in the refinement in the riding model approximation, with *U*_iso_(H) set to 1.2*U*_eq_(C). The O-bound and N-bound H-atoms were located in difference-Fourier maps but were refined with distance restraints of O—H = 0.84±0.01 Å and N—H = 0.88±0.01 Å, and with *U*_iso_(H) set to 1.5*U*_eq_(O) and 1.2*U*_eq_(N). The maximum and minimum residual electron density peaks of 1.15 and 0.69 e Å^−3^, respectively, were located 0.90 and 0.87 Å from the Cd^II^ cation.

## Supplementary Material

Crystal structure: contains datablock(s) I, global. DOI: 10.1107/S2056989017011677/hb7697sup1.cif

Structure factors: contains datablock(s) I. DOI: 10.1107/S2056989017011677/hb7697Isup2.hkl

CCDC reference: 1446968

Additional supporting information:  crystallographic information; 3D view; checkCIF report

## Figures and Tables

**Table 1:** Selected bond lengths (Å)

Cd—O1	2.3448 (17)	Cd—N2	2.398 (2)
Cd—O2	2.3560 (16)	Cd—Cl1	2.5283 (6)
Cd—N1	2.448 (2)	Cd—O1*W*	2.2265 (18)

**Table 2:** Hydrogen-bond geometry (Å, °)

*D*—H⋯*A*	*D*—H	H⋯*A*	*D*⋯*A*	*D*—H⋯*A*
O1*W*—H1*W*⋯O1^i^	0.83 (2)	1.90 (2)	2.728 (2)	176 (3)
O1*W*—H2*W*⋯O2^ii^	0.83 (1)	1.84 (1)	2.670 (2)	177 (3)
N1—H1*N*⋯Cl1^i^	0.88 (2)	2.57 (2)	3.428 (2)	166 (2)
N1—H2*N*⋯Cl1^iii^	0.88 (2)	2.52 (2)	3.374 (2)	165 (2)
N2—H3*N*⋯Cl1^iii^	0.87 (2)	2.53 (2)	3.385 (2)	168 (2)
N2—H4*N*⋯Cl1^iv^	0.88 (2)	2.52 (2)	3.322 (2)	153 (3)

Symmetry codes: (i) 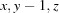; (ii) 
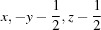
; (iii) 
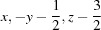
; (iv) 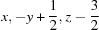.

**Table 3:** Percentage contributions of inter-atomic contacts to the Hirshfeld surfaces for (I)

Contact	Percentage contribution
H⋯H	45.4
O⋯H/H⋯O	22.9
Cl⋯H/H⋯Cl	19.0
C⋯H/H⋯C	11.2
C⋯Cl/Cl⋯C	0.7
C⋯C	0.4
Cl⋯O/O⋯Cl	0.3
Cd⋯H/H⋯Cd	0.1

**Table 4:** Summary of short inter-atomic contacts (Å) in (I)

Contact	Distance	Symmetry operation
H1*W*⋯H3*A*	2.32	*x*, −1 + *y*, *z*
H8⋯H8	2.38	1 − *x*, − *y*, 1 − *z*
O1*W*⋯H3*A*	2.64	*x*, − 1 + *y*, *z*

**Table 5:** Experimental details

Crystal data	
Chemical formula	[Cd_2_Cl_2_(C_6_H_8_O_4_)(C_6_H_8_N_2_)_2_(H_2_O)_2_]
*M*_r_	692.14
Crystal system, space group	Monoclinic, *P*2_1_/*c*
Temperature (K)	100
*a*, *b*, *c* (Å)	20.4710 (8), 5.5578 (2), 10.7910 (3)
β (°)	98.122 (3)
*V* (Å^3^)	1215.42 (7)
*Z*	2
Radiation type	Mo *K*α
μ (mm^−1^)	2.01
Crystal size (mm)	0.33 × 0.22 × 0.10

Data collection	
Diffractometer	Agilent Technologies SuperNova Dual diffractometer with Atlas detector
Absorption correction	Multi-scan (*CrysAlis PRO*; Agilent, 2013 ▸)
*T*_min_, *T*_max_	0.842, 1.000
No. of measured, independent and observed [*I* > 2σ(*I*)] reflections	15862, 3393, 2992
*R*_int_	0.039
(sin θ/λ)_max_ (Å^−1^)	0.710

Refinement	
*R*[*F* ^2^ > 2σ(*F* ^2^)], *wR*(*F* ^2^), *S*	0.028, 0.065, 1.04
No. of reflections	3393
No. of parameters	163
No. of restraints	6
H-atom treatment	H atoms treated by a mixture of independent and constrained refinement
Δρ_max_, Δρ_min_ (e Å^−3^)	1.15, −0.69

Computer programs: *CrysAlis PRO* (Agilent, 2013 ▸), *SHELXS97* (Sheldrick, 2008 ▸), *SHELXL2014* (Sheldrick, 2015 ▸), *ORTEP-3 for Windows* (Farrugia, 2012 ▸), *DIAMOND* (Brandenburg, 2006 ▸) and *publCIF* (Westrip, 2010 ▸).

## supplementary crystallographic information

### Crystal data

**Table:**

[Cd_2_Cl_2_(C_6_H_8_O_4_)(C_6_H_8_N_2_)_2_(H_2_O)_2_]	*F*(000) = 684
*M**_r_* = 692.14	*D*_x_ = 1.891 Mg m^−^^3^
Monoclinic, *P*2_1_/*c*	Mo *K*α radiation, λ = 0.71073 Å
*a* = 20.4710 (8) Å	Cell parameters from 7493 reflections
*b* = 5.5578 (2) Å	θ = 3.8–30.0°
*c* = 10.7910 (3) Å	µ = 2.01 mm^−^^1^
β = 98.122 (3)°	*T* = 100 K
*V* = 1215.42 (7) Å^3^	Prism, brown
*Z* = 2	0.33 × 0.22 × 0.10 mm

### Data collection

**Table:**

Agilent Technologies SuperNova Dual diffractometer with Atlas detector	3393 independent reflections
Radiation source: SuperNova (Mo) X-ray Source	2992 reflections with *I* > 2σ(*I*)
Mirror monochromator	*R*_int_ = 0.039
Detector resolution: 10.4041 pixels mm^-1^	θ_max_ = 30.3°, θ_min_ = 3.0°
ω scan	*h* = −28→26
Absorption correction: multi-scan (CrysAlis PRO; Agilent, 2013)	*k* = −7→7
*T*_min_ = 0.842, *T*_max_ = 1.000	*l* = −14→14
15862 measured reflections	

### Refinement

**Table:**

Refinement on *F*^2^	6 restraints
Least-squares matrix: full	H atoms treated by a mixture of independent and constrained refinement
*R*[*F*^2^ > 2σ(*F*^2^)] = 0.028	*w* = 1/[σ^2^(*F*_o_^2^) + (0.0312*P*)^2^ + 0.8274*P*] where *P* = (*F*_o_^2^ + 2*F*_c_^2^)/3
*wR*(*F*^2^) = 0.065	(Δ/σ)_max_ < 0.001
*S* = 1.04	Δρ_max_ = 1.15 e Å^−^^3^
3393 reflections	Δρ_min_ = −0.69 e Å^−^^3^
163 parameters	

### Special details

**Table:**

Geometry. All esds (except the esd in the dihedral angle between two l.s. planes) are estimated using the full covariance matrix. The cell esds are taken into account individually in the estimation of esds in distances, angles and torsion angles; correlations between esds in cell parameters are only used when they are defined by crystal symmetry. An approximate (isotropic) treatment of cell esds is used for estimating esds involving l.s. planes.

### Fractional atomic coordinates and isotropic or equivalent isotropic displacement parameters (Å^2^)

**Table:**

	*x*	*y*	*z*	*U*_iso_*/*U*_eq_	
Cd	0.20549 (2)	0.38825 (3)	0.25421 (2)	0.01173 (6)	
Cl1	0.26037 (3)	0.56615 (11)	0.45833 (5)	0.01653 (13)	
O1	0.14096 (9)	0.7293 (3)	0.19608 (15)	0.0172 (4)	
O2	0.13895 (9)	0.4366 (3)	0.05909 (15)	0.0155 (4)	
O1W	0.12696 (9)	0.1564 (3)	0.31419 (16)	0.0155 (4)	
H1W	0.1293 (15)	0.026 (3)	0.277 (3)	0.023*	
H2W	0.1321 (15)	0.127 (5)	0.3906 (11)	0.023*	
N1	0.28158 (11)	0.0464 (4)	0.26867 (19)	0.0149 (4)	
H1N	0.2813 (14)	−0.063 (4)	0.327 (2)	0.018*	
H2N	0.2722 (14)	−0.009 (5)	0.1919 (14)	0.018*	
N2	0.29273 (11)	0.4407 (4)	0.12990 (19)	0.0145 (4)	
H3N	0.2803 (13)	0.324 (4)	0.078 (2)	0.017*	
H4N	0.2981 (14)	0.582 (3)	0.097 (3)	0.017*	
C1	0.12011 (12)	0.6431 (5)	0.0893 (2)	0.0147 (5)	
C2	0.07303 (14)	0.7824 (6)	−0.0037 (2)	0.0240 (6)	
H2A	0.0485	0.6667	−0.0627	0.029*	
H2B	0.0991	0.8867	−0.0526	0.029*	
C3	0.02372 (12)	0.9371 (5)	0.0503 (2)	0.0165 (5)	
H3A	0.0476	1.0597	0.1059	0.020*	
H3B	−0.0018	0.8357	0.1017	0.020*	
C4	0.34453 (12)	0.1628 (4)	0.2844 (2)	0.0133 (5)	
C5	0.35047 (13)	0.3687 (4)	0.2120 (2)	0.0146 (5)	
C6	0.40716 (12)	0.5067 (5)	0.2319 (2)	0.0169 (5)	
H6	0.4112	0.6456	0.1822	0.020*	
C7	0.45817 (13)	0.4418 (5)	0.3248 (2)	0.0202 (5)	
H7	0.4972	0.5360	0.3387	0.024*	
C8	0.45193 (13)	0.2395 (5)	0.3971 (2)	0.0210 (5)	
H8	0.4866	0.1973	0.4616	0.025*	
C9	0.39589 (13)	0.0984 (5)	0.3766 (2)	0.0177 (5)	
H9	0.3925	−0.0421	0.4254	0.021*	

### Atomic displacement parameters (Å^2^)

**Table:**

	*U*^11^	*U*^22^	*U*^33^	*U*^12^	*U*^13^	*U*^23^
Cd	0.01482 (10)	0.00958 (9)	0.01025 (9)	0.00074 (6)	−0.00008 (6)	0.00069 (6)
Cl1	0.0255 (3)	0.0120 (3)	0.0112 (2)	−0.0020 (2)	−0.0006 (2)	−0.0006 (2)
O1	0.0224 (9)	0.0157 (9)	0.0127 (8)	0.0044 (7)	−0.0005 (7)	−0.0009 (7)
O2	0.0200 (9)	0.0150 (9)	0.0111 (8)	0.0031 (7)	0.0007 (7)	−0.0010 (6)
O1W	0.0210 (9)	0.0138 (9)	0.0114 (8)	−0.0019 (7)	0.0015 (7)	0.0002 (7)
N1	0.0212 (11)	0.0123 (10)	0.0109 (9)	−0.0019 (8)	0.0014 (8)	0.0008 (8)
N2	0.0214 (11)	0.0097 (10)	0.0119 (9)	−0.0005 (8)	0.0007 (8)	0.0027 (8)
C1	0.0156 (12)	0.0162 (12)	0.0123 (11)	0.0028 (9)	0.0027 (9)	0.0018 (9)
C2	0.0267 (14)	0.0310 (16)	0.0133 (11)	0.0147 (12)	0.0000 (10)	0.0009 (11)
C3	0.0189 (13)	0.0156 (12)	0.0140 (11)	0.0057 (10)	−0.0018 (9)	0.0008 (9)
C4	0.0161 (12)	0.0124 (11)	0.0117 (10)	0.0026 (9)	0.0025 (9)	−0.0016 (9)
C5	0.0185 (12)	0.0145 (12)	0.0112 (11)	0.0027 (9)	0.0027 (9)	−0.0012 (9)
C6	0.0206 (13)	0.0132 (12)	0.0174 (12)	0.0010 (10)	0.0047 (9)	−0.0011 (9)
C7	0.0164 (13)	0.0221 (14)	0.0222 (13)	−0.0039 (10)	0.0038 (10)	−0.0035 (11)
C8	0.0173 (12)	0.0259 (15)	0.0188 (12)	0.0061 (11)	−0.0010 (9)	−0.0014 (10)
C9	0.0221 (13)	0.0165 (13)	0.0148 (12)	0.0042 (10)	0.0034 (10)	0.0014 (9)

### Geometric parameters (Å, º)

**Table:**

Cd—O1	2.3448 (17)	C2—C3	1.505 (4)
Cd—O2	2.3560 (16)	C2—H2A	0.9900
Cd—N1	2.448 (2)	C2—H2B	0.9900
Cd—N2	2.398 (2)	C3—C3^i^	1.521 (5)
Cd—Cl1	2.5283 (6)	C3—H3A	0.9900
Cd—O1W	2.2265 (18)	C3—H3B	0.9900
O1—C1	1.265 (3)	C4—C9	1.389 (3)
O2—C1	1.268 (3)	C4—C5	1.400 (3)
O1W—H1W	0.834 (10)	C5—C6	1.382 (4)
O1W—H2W	0.832 (10)	C6—C7	1.389 (4)
N1—C4	1.430 (3)	C6—H6	0.9500
N1—H1N	0.875 (10)	C7—C8	1.385 (4)
N1—H2N	0.879 (10)	C7—H7	0.9500
N2—C5	1.431 (3)	C8—C9	1.381 (4)
N2—H3N	0.871 (10)	C8—H8	0.9500
N2—H4N	0.874 (10)	C9—H9	0.9500
C1—C2	1.504 (3)		
			
O1W—Cd—O1	98.22 (6)	O2—C1—C2	118.9 (2)
O1W—Cd—O2	88.62 (6)	O1—C1—Cd	59.78 (12)
O1—Cd—O2	55.66 (6)	O2—C1—Cd	60.29 (12)
O1W—Cd—N2	149.12 (7)	C2—C1—Cd	179.18 (18)
O1—Cd—N2	100.84 (7)	C1—C2—C3	116.0 (2)
O2—Cd—N2	82.44 (7)	C1—C2—H2A	108.3
O1W—Cd—N1	90.66 (7)	C3—C2—H2A	108.3
O1—Cd—N1	166.89 (6)	C1—C2—H2B	108.3
O2—Cd—N1	115.34 (6)	C3—C2—H2B	108.3
N2—Cd—N1	67.19 (7)	H2A—C2—H2B	107.4
O1W—Cd—Cl1	102.90 (5)	C2—C3—C3^i^	112.5 (3)
O1—Cd—Cl1	94.65 (4)	C2—C3—H3A	109.1
O2—Cd—Cl1	149.72 (5)	C3^i^—C3—H3A	109.1
N2—Cd—Cl1	99.52 (5)	C2—C3—H3B	109.1
N1—Cd—Cl1	92.72 (5)	C3^i^—C3—H3B	109.1
C1—O1—Cd	92.43 (14)	H3A—C3—H3B	107.8
C1—O2—Cd	91.84 (14)	C9—C4—C5	119.6 (2)
Cd—O1W—H1W	106 (2)	C9—C4—N1	123.1 (2)
Cd—O1W—H2W	114 (2)	C5—C4—N1	116.8 (2)
H1W—O1W—H2W	107 (3)	C6—C5—C4	120.3 (2)
C4—N1—Cd	102.19 (15)	C6—C5—N2	122.9 (2)
C4—N1—H1N	109.0 (19)	C4—C5—N2	116.4 (2)
Cd—N1—H1N	121 (2)	C5—C6—C7	119.8 (2)
C4—N1—H2N	110.0 (19)	C5—C6—H6	120.1
Cd—N1—H2N	99 (2)	C7—C6—H6	120.1
H1N—N1—H2N	115 (3)	C8—C7—C6	119.8 (2)
C5—N2—Cd	103.59 (14)	C8—C7—H7	120.1
C5—N2—H3N	109 (2)	C6—C7—H7	120.1
Cd—N2—H3N	95.4 (19)	C9—C8—C7	120.8 (2)
C5—N2—H4N	111.2 (19)	C9—C8—H8	119.6
Cd—N2—H4N	119 (2)	C7—C8—H8	119.6
H3N—N2—H4N	117 (3)	C8—C9—C4	119.7 (2)
O1—C1—O2	120.1 (2)	C8—C9—H9	120.1
O1—C1—C2	121.0 (2)	C4—C9—H9	120.1
			
Cd—O1—C1—O2	0.2 (2)	C9—C4—C5—N2	−172.4 (2)
Cd—O1—C1—C2	−179.7 (2)	N1—C4—C5—N2	0.0 (3)
Cd—O2—C1—O1	−0.2 (2)	Cd—N2—C5—C6	−130.3 (2)
Cd—O2—C1—C2	179.7 (2)	Cd—N2—C5—C4	42.1 (2)
O1—C1—C2—C3	−34.4 (4)	C4—C5—C6—C7	−0.5 (4)
O2—C1—C2—C3	145.7 (3)	N2—C5—C6—C7	171.6 (2)
C1—C2—C3—C3^i^	−177.6 (3)	C5—C6—C7—C8	−0.1 (4)
Cd—N1—C4—C9	131.4 (2)	C6—C7—C8—C9	1.1 (4)
Cd—N1—C4—C5	−40.8 (2)	C7—C8—C9—C4	−1.5 (4)
C9—C4—C5—C6	0.2 (4)	C5—C4—C9—C8	0.8 (4)
N1—C4—C5—C6	172.6 (2)	N1—C4—C9—C8	−171.1 (2)

Symmetry code: (i) −*x*, −*y*+2, −*z*.

### Hydrogen-bond geometry (Å, º)

**Table:**

*D*—H···*A*	*D*—H	H···*A*	*D*···*A*	*D*—H···*A*
O1*W*—H1*W*···O1^ii^	0.83 (2)	1.90 (2)	2.728 (2)	176 (3)
O1*W*—H2*W*···O2^iii^	0.83 (1)	1.84 (1)	2.670 (2)	177 (3)
N1—H1*N*···Cl1^ii^	0.88 (2)	2.57 (2)	3.428 (2)	166 (2)
N1—H2*N*···Cl1^iv^	0.88 (2)	2.52 (2)	3.374 (2)	165 (2)
N2—H3*N*···Cl1^iv^	0.87 (2)	2.53 (2)	3.385 (2)	168 (2)
N2—H4*N*···Cl1^v^	0.88 (2)	2.52 (2)	3.322 (2)	153 (3)

Symmetry codes: (ii) *x*, *y*−1, *z*; (iii) *x*, −*y*−1/2, *z*−1/2; (iv) *x*, −*y*−1/2, *z*−3/2; (v) *x*, −*y*+1/2, *z*−3/2.
